# Slow-cycling stem cells in hydra contribute to head regeneration

**DOI:** 10.1242/bio.201410512

**Published:** 2014-11-28

**Authors:** Niraimathi Govindasamy, Supriya Murthy, Yashoda Ghanekar

**Affiliations:** Institute for Stem Cell Biology and Regenerative Medicine (inStem), National Centre for Biological Sciences, GKVK Campus, Bellary Road, Bangalore 560065, India

**Keywords:** Hydra, Quiescence, Regeneration, Stem cells, Cnidaria

## Abstract

Adult stem cells face the challenge of maintaining tissue homeostasis by self-renewal while maintaining their proliferation potential over the lifetime of an organism. Continuous proliferation can cause genotoxic/metabolic stress that can compromise the genomic integrity of stem cells. To prevent stem cell exhaustion, highly proliferative adult tissues maintain a pool of quiescent stem cells that divide only in response to injury and thus remain protected from genotoxic stress. Hydra is a remarkable organism with highly proliferative stem cells and ability to regenerate at whole animal level. Intriguingly, hydra does not display consequences of high proliferation, such as senescence or tumour formation. In this study, we investigate if hydra harbours a pool of slow-cycling stem cells that could help prevent undesirable consequences of continuous proliferation. Hydra were pulsed with the thymidine analogue 5-ethynyl-2′-deoxyuridine (EdU) and then chased in the absence of EdU to monitor the presence of EdU-retaining cells. A significant number of undifferentiated cells of all three lineages in hydra retained EdU for about 8–10 cell cycles, indicating that these cells did not enter cell cycle. These label-retaining cells were resistant to hydroxyurea treatment and were predominantly in the G2 phase of cell cycle. Most significantly, similar to mammalian quiescent stem cells, these cells rapidly entered cell division during head regeneration. This study shows for the first time that, contrary to current beliefs, cells in hydra display heterogeneity in their cell cycle potential and the slow-cycling cells in this population enter cell cycle during head regeneration. These results suggest an early evolution of slow-cycling stem cells in multicellular animals.

## INTRODUCTION

Hydra has been used as a model system to study development, regeneration and evolutionary studies for many years. Hydra has an extraordinary ability to regenerate; when cut into pieces, each piece of the body column can regenerate into an adult animal within 3–4 days, with the regenerated animal maintaining its original polarity (Bosch, 2003; Watanabe et al., 2009). It can also regenerate from a cluster of dissociated single cells in which the axis has been disrupted; this cluster of cells undergoes *de novo* patterning to give rise to a well-developed adult organism (Gierer et al., 1972). Cells in an adult hydra thus preserve the ability to respond to morphogenetic signals and undergo patterning in a manner similar to embryonic stem cells. Hydra does not exhibit senescence under laboratory conditions (Martínez, 1998). Hydra is thus considered to be an immortal organism with infinite regenerative ability.

Hydra consists of three cell lineages; ectodermal epithelial lineage, endodermal epithelial lineage and interstitial lineage. Ectodermal epithelial cells form the outer layer of body column and endodermal cells form the inner digestive layer. Cells from both ectodermal and endodermal lineages differentiate into specialized cells at the two extremities. The two layers are separated by an acellular extracellular matrix called mesoglea (Sarras, 2012). Interstitial cells are dispersed in the spaces between ectodermal and endodermal cells. Interstitial lineage gives rise to somatic cells such as stinging cells or nematocytes, neurons, gland cells and germ cells. The three lineages do not interconvert (Hobmayer et al., 2012; Wittlieb et al., 2006). The cells in body column proliferate and are continuously displaced towards hypostome and foot. The cells differentiate in response to positional information in the body column as they migrate and finally slough off (Campbell, 1973).

The stem cells in ectodermal and endodermal lineages are considered to be “multifunctional stem cells”. These cells are epitheliomuscular cells with morphology and functions of well-developed epithelial cells and contractile function similar to muscle cells but also retain the ability to self-renew and differentiate (Hobmayer et al., 2012; Watanabe et al., 2009). Epithelial cells divide once every 3–4 days (David and Campbell, 1972) and all epithelial cells in the gastric region are considered to be stem cells (Bosch et al., 2010; Wittlieb et al., 2006). Stem cells of the interstitial lineage on the other hand are better defined and are multipotent stem cells that give rise to both somatic and germ cells (David, 2012). These can be identified by their morphology and occur either as single cells (1s) or in pairs (2s). Interstitial cells divide with a cell cycle time of 16–27 hours (Campbell and David, 1974). The ability of stem cells in hydra to divide and differentiate appears to be unlimited, since hydra does not show senescence.

This ability of hydra stem cells to undergo continuous self-renewal/differentiation over many years is in complete contrast to adult stem cells in higher organisms which lose their proliferative potential with time. As an organism ages, there is a decline in the ability of adult stem cells in tissues to maintain homeostasis and to repair damage caused due to injury (Cheung and Rando, 2013; Rossi et al., 2008). An important factor contributing to this loss of proliferative potential is genotoxic stress such as mutations acquired during replication and shortening of telomeres during each cell cycle. Adult stem cells thus need to preserve the ability to self-renew while undergoing continuous proliferations for normal homeostasis, especially in highly dividing systems such as hematopoietic cells or intestinal epithelial cells. To protect stem cells from errors of replication, adult tissues have a pool of slow-cycling or quiescent stem cells that do not undergo cell division under normal physiological conditions and remain quiescent in G0 phase of cell cycle (Cheung and Rando, 2013; Fuchs, 2009). These cells either divide at a very slow rate or divide only in response to tissue damage, ensuring that cells contributing to regeneration have better genomic integrity. Such slow-cycling cells are identified by their ability to retain thymidine analog such as bromodeoxyuridine (BrdU) over long periods of time and hence are also called “label-retaining cells”. Quiescent cells have been observed in several mammalian systems such as limbal stem cells (Cotsarelis et al., 1989), hair follicle stem cells (Morris and Potten, 1999), intestinal stem cells (Cheshier et al., 1999) and hematopoietic stem cells (Wilson et al., 2008). Prior to identification of specific markers, stem cells in adult tissues were in fact identified by their ability to retain thymidine analogs, indicating their ability to remain quiescent (Fuchs and Horsley, 2011). Although label retention is no longer considered the hallmark of stem cells, quiescence is an important feature of adult stem cells and the loss of quiescent cells has an adverse effect on the long-term proliferation potential of stem cells (Cheng et al., 2000a; Cheng et al., 2000b). Label-retaining cells have also been observed in invertebrates such as locusts (Illa-Bochaca and Montuenga, 2006), larval silk worm *Bombyx mori* (Tan et al., 2013) and in neoblasts (adult multipotent stem cells) of the platyhelminth *Macrostomum* (Verdoodt et al., 2012). The functional relevance of label-retaining cells in invertebrates remains unclear.

Considering the highly proliferative nature of hydra cells, it is interesting to note that hydra does not show any effect of genotoxicity. In this study we carried out pulse–chase studies with thymidine analog 5-ethynyl-2′-deoxyuridine (EdU) to investigate if hydra harbours slow-cycling stem cells. Our studies show that stem cells from all three lineages in hydra indeed have a pool of stem cells that do not divide frequently and are paused in G2. These cells can remain undivided for 8–10 cell cycles while retaining their ability to undergo cell division. Most importantly during head regeneration, these slow-cycling cells are recruited to divide within one hour post-amputation and contribute to head regeneration, resembling quiescent stem cells from mammalian adult tissues.

## MATERIALS AND METHODS

### Hydra culture

*Hydra magnipapillata* was cultured in hydra medium (0.1 mM KCl, 1 mM CaCl_2_, 1 mM NaCl, 0.1 mM MgSO_4_, 1 mM Tris-Cl, pH 8.0) at 18°C as described earlier (Krishna et al., 2013). Hydra polyps were fed on alternate days with freshly hatched *Artemia* nauplii. *H. vulgaris* AEP expressing GFP under *nanos1* promoter specific for 1s and 2s interstitial cells (cnnos1::eGFP) was a kind gift from Professor Bosch, University of Kiel, Germany and was cultured in a similar manner.

### EdU pulse and chase

5-ethynyl-2′-deoxyuridine EdU (Invitrogen) can efficiently label cycling cells in hydra as seen with a short pulse of three hours (supplementary material Fig. S1A), during which EdU was rapidly incorporated in dividing cells in the body column. For EdU pulse, approximately 50 regularly fed animals were pulsed with 2 mM EdU for one week in 3 ml hydra medium or with 1 mM EdU in hydra medium for four weeks. EdU was changed every 48 h. To check if the labelling efficiency of EdU changes during this incubation, hydra were labelled with 2 mM EdU for 24 h. The spent medium of these pulsed hydra was added to another set of hydra for 24 h. The labelling index obtained using fresh EdU and EdU from spent medium was comparable (data not shown), indicating that EdU is not depleted from medium during 48 hours of incubation. During pulse, hydra were fed with freshly hatched *Artemia* nauplii every other day and fresh EdU was added after every feed. During chase, buds were removed from cultures as soon as they fell off the parent and polyps were fed every other day. For EdU and Bromodeoxyuridine (BrdU) colocalization, hydra in chase were incubated with 2 mM BrdU (Roche) for 3–7 days. This concentration of BrdU was sufficient for detection of incorporated BrdU with this kit.

### Detection of EdU in whole animals and macerates

EdU staining was performed either in whole animals or in hydra macerates. Whole animals were relaxed in 2% urethane, fixed in 3.7% paraformaldehyde (PFA) and Click-iT® reaction was carried out as per manufacturer's instructions using Alexa 488 or Alexa 647 azide. Animals that were not pulsed with EdU were used as negative control and did not exhibit any signal after Click-iT® reaction. To monitor EdU in different cell types, maceration of hydra polyps in chase was performed as described (David, 1973). Five hydra in chase were macerated, macerates were fixed with 3.7% PFA and stained for EdU as described earlier. Nuclei were stained with 4′,6-diamidino-2-phenylindole (DAPI). Polyps or macerates were mounted in mowiol and imaging was performed using BX53 microscope from Olympus using cellSens Standard Image Acquisition software. The cells were identified based on their morphology under 40× phase contrast microscope as described earlier (David, 1973). To find out the percentage of cells retaining EdU, only cells in which EdU completely colocalized with DAPI were considered (supplementary material Fig. S1B). The experiments were performed several times and data from three independent experiments are shown. The number of cells counted were as follows: 871–1331 ectodermal epithelial cells, 634–716 endodermal epithelial cells, 522–896 1s/2s cells and 432–634 gland cells.

### Immunofluorescence

For co-staining, Click-iT® reaction was performed after immunostaining since Click-iT® reaction interferes with immunostaining. For detection of BrdU and EdU colocalization, immunostaining for BrdU was performed using Roche BrdU labelling and staining kit, followed by Click-iT® reaction. The BrdU antibody from Roche kit did not cross-react with EdU, as seen by lack of immunostaining in polyps labelled with EdU but not with BrdU (data not shown). Similarly immunostaining for phospho-histone H3 (S10) was performed using anti-H3P antibody from Abcam (ab14955) followed by Click-iT® reaction for EdU detection. The nuclei were stained with DAPI and macerates were mounted using mowiol.

### Localization of label-retaining cells

To observe localization of label-retaining cells with respect to the mesoglea, cnnos1::eGFP transgenic line, which expresses GFP at high level in 1s/2s cells was used. This line was pulsed and chased for one week as described earlier and experiment was performed at one week of chase. Click-iT® reaction results in loss of fluorescence from GFP and therefore GFP was detected by immunostaining. Immunofluorescence was performed with anti-GFP antibody ab290 from Abcam and anti-laminin antibody raised to hydra laminin, a gift from Professor Zhang, University of Kansas, Kansas, USA, before performing Click-iT® reaction. Imaging to check localization of label-retaining cells with respect to laminin was performed using Zeiss LSM 700 confocal microscope. To quantitate the number of label-retaining cells close to extracellular matrix, cells with high GFP expression were selected. Among these GFP-expressing cells, 50 cells each with complete and partial EdU were counted and their localization close to laminin was checked by scanning sections of confocal images.

### Hydroxyurea treatment

Hydra were pulsed with EdU for one week as described above, chased for two days without EdU and were then treated with 10 mM hydroxyurea in hydra medium for five consecutive days. This treatment leads to more than 95% reduction in the number of interstitial cells (data not shown). Hydra were macerated at the end of treatment as well as at one day and at three days after hydroxyurea treatment was over and stained for EdU. Data from two independent experiments are shown.

### Measurement of nuclear DNA content of slow-cycling cells

For measurement of nuclear DNA content in interstitial cells, polyps at one week of chase were used and for epithelial cells polyps at 3.5 weeks of chase were used. Five polyps were macerated, stained for EdU and saturating concentration of DAPI (10 µg/ml). Cells were imaged with cellSens Standard Image Acquisition software and the fluorescence was measured using ImageJ software. The intensity of fluorescence of nuclear DNA of neurons, 1s/2s cells and epithelial cells that retained label and those that did not retain label was calculated after subtracting background intensity. The integrated intensity was divided by average integrated intensity of neuronal cells to obtain ratio of DNA content of each cell with respect to the DNA content of neurons. Most of the neurons had ratio of 1 which was taken as 2N DNA content. Ratio between 1 and 2 was taken to represent cells in S phase, while that between 2–3 was taken as G2 phase. Few cells with ratio higher than 3 were observed but were excluded from further analysis. The data represented here are pooled from three independent experiments.

### Regeneration assay

To investigate the role of slow-cycling interstitial cells, hydra at one week of chase were used and to investigate the role of slow-cycling epithelial cells, hydra at 2.5–3.5 weeks of chase were used for the experiment. Fifteen animals were used for each time point. The animals were cut in the midgastric region and the lower foot region, which develops into head, was allowed to regenerate for one hour and three hours at 18°C. At the end of incubation, the regenerating tips from lower halves were collected (∼500 µm) and macerated for EdU staining. As control, animals were cut in the midgastric region and the tips of the lower half were macerated immediately. The experiment was performed several times and the data from three independent experiments are presented for epithelial cells and data from two independent experiments are shown for interstitial cells.

## RESULTS

### Hydra harbours a population of label-retaining cells

**Fig. 1.**
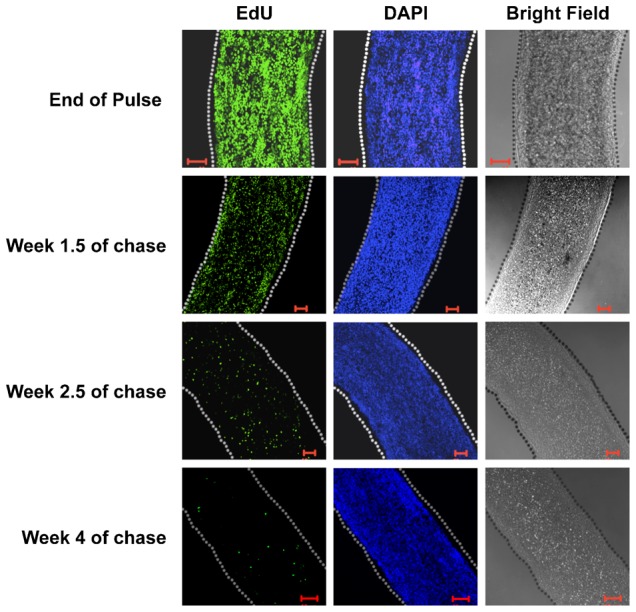
Label-retaining cells in body column of hydra at different times of chase. Hydra were pulsed with EdU for one week and then cultured without EdU for four weeks. At the end of pulse, the entire body column was labelled as expected. As chase progressed from one week to four weeks, the number of label-retaining cells decreased but at the end of four weeks, label-retaining cells were observed in the body column. Images of hypostome and foot at different times of chase are shown in supplementary material Fig. S2A,B. Scale bars: 50 µm.

Studies on proliferation kinetics of hydra have shown that the cell cycle of epithelial cells is 3–4 days (David and Campbell, 1972) while that of interstitial cells is 16–27 hours (Campbell and David, 1974). Gland cells can also undergo division with a cell cycle time of about 64–83 hours (Schmidt and David, 1986). To check if hydra has any label-retaining cells, hydra polyps were pulsed with EdU for one week to ensure all cells undergo cell division at least twice during the pulse and then chased in medium without EdU. Label retention was investigated over next 4–5 weeks. In frequently dividing cells, EdU gets diluted and is eventually lost from cells. The cells that do not divide, such as differentiated cells and stem cells that might have entered quiescence after EdU incorporation, retain EdU during chase. During chase hydra were incubated in normal hydra medium, fed every other day and buds were removed from cultures as soon as they fell off. Pulsed hydra were able to bud and regenerate at a rate comparable to the control animals that were not pulsed, indicating that EdU did not have any toxic effect on hydra (data not shown). As shown in Fig. 1 and supplementary material Fig. S2, at the end of one week pulse, cells throughout the polyp were labelled with EdU, as would be expected from hydra cell kinetics. With continued chase, the number of cells with EdU decreased, either through loss of cells from the polyps or due to division of labelled stem cells (Fig. 1). However a small but significant number of EdU-positive cells could be detected in the body column and hypostome at the end of four weeks (Fig. 1; supplementary material Fig. S2). The presence of EdU-retaining cells was not surprising in the areas with high number of differentiated cells such as hypostome; the cells in hypostome have a very slow turnover and the label-retaining cells in hypostome could have differentiated soon after incorporating EdU (supplementary material Fig. S2). The presence of EdU-retaining cells in the body column, typically believed to consist of continuously dividing stem cells, was contrary to the current understanding about hydra cell kinetics. Slow-cycling cells present at the end of chase were present throughout the body column and did not localise to any particular area of polyp.

**Fig. 2.**
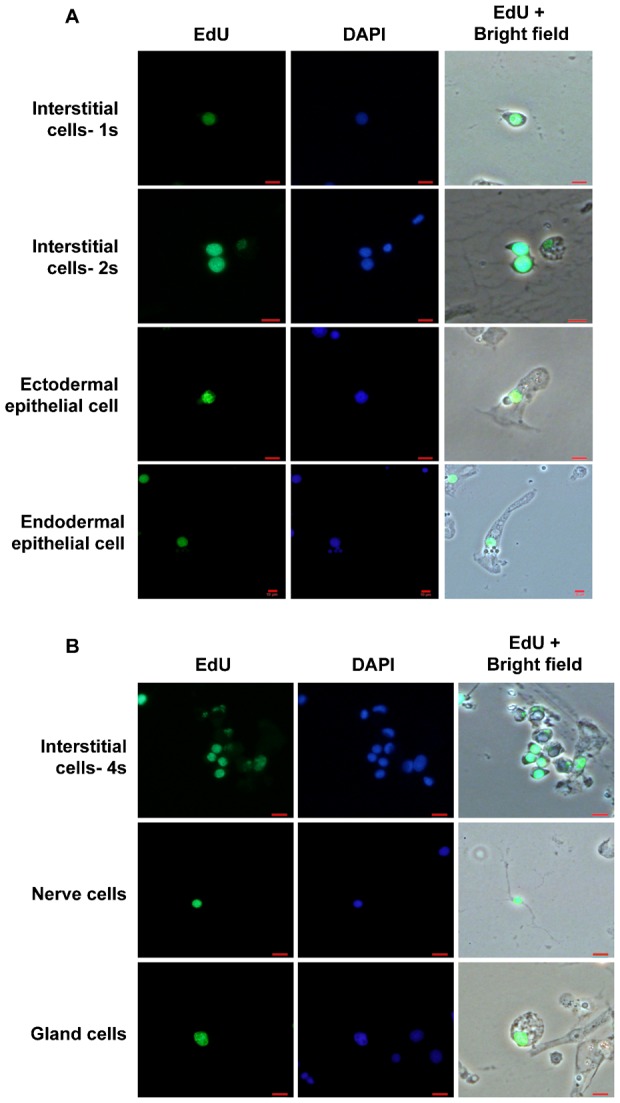
Identification of label-retaining cells. (A) Label-retaining ectodermal and endodermal epithelial cells at the end of four weeks and 1s/2s interstitial cells at the end of one week. (B) EdU retention was also observed in differentiated cells like gland cells and neurons at the end of four weeks and in 4s nematoblasts at the end of one week of chase. Scale bars: 10 µm.

Hydra were simultaneously macerated to identify the cell types that retained label, as described (David, 1973). The cells retaining label were defined as those which had EdU signal that completely colocalized with signal from DAPI staining of nuclear DNA. Cells with partial/punctate EdU signal were not considered to be label-retaining cells since dilution of signal indicates that cells have undergone cell division (supplementary material Fig. S1B). The morphology of label-retaining nuclei and cells was normal (Fig. 2). After one week of pulse, the labelling index of interstitial cells was approximately 94–98%. The labelling index of epithelial cells was much lower; the labelling index of ectodermal epithelial cells varied between 54–80% and that of endodermal cells varied between 41–56% (Table 1). The labelling index was not 100% in any cell lineage indicating lack of cell division in a substantial number of cells, especially in the epithelial lineages. The fact that a fraction of stem cells of all lineages remained undivided suggested that morphologically similar cells indeed display variation in the frequency of cell division and not all cells in hydra undergo incessant cell division. A longer pulse of four weeks was also performed to determine if this time of pulse is sufficient to label all cells capable of undergoing cell division. Upon longer pulse of four weeks, the labelling index increased; all 1s and 2s interstitial cells were completely labelled. The labelling index of ectodermal and endodermal epithelial cells was >90% but not 100%, indicating that a few epithelial cells remained undivided even after 4 weeks (Table 1). A similar labelling index was also observed when regenerating aggregates of hydra were pulsed with EdU. The aggregates of dissociated single cells (Gierer et al., 1972) were pulsed with EdU from the time aggregates were generated until hydranths developed and fell off the aggregate (data not shown). Long pulse with EdU, of both whole animals and developing aggregates, however adversely affected hydra which showed defects such as slow rate of budding and tentacle formation. Subsequent studies were therefore performed with one week pulse.

**Table 1.**
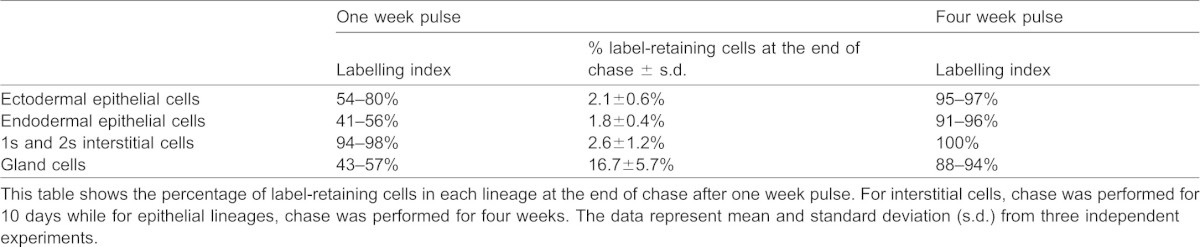
Labelling index at the end of one week and four week pulse

After one week of pulse with EdU, until ten days of chase, undifferentiated cells from all the three lineages retained EdU. At the end of 10 days, 2.6±1.2% 1s and 2s cells with complete label were detected. Representative picture of cells at 1 week are shown in Fig. 2A. These cells could be detected until 10 days after which only partial labelled interstitial cells were detected. Ectodermal and endodermal epithelial cells retained label longer, for four weeks and sometimes until five weeks. At four weeks of chase, 2.1±0.6% ectodermal epithelial cells and 1.8±0.4% endodermal epithelial cells had complete EdU label (Table 1 and Fig. 2A). Among differentiated cells, nematoblasts retaining label could be detected up to two weeks while a significant number of neurons and gland cells could be detected up to four weeks of chase (Fig. 2B). Considering that cell cycle time of epithelial cells is 3–4 days and that of interstitial cells is 18–27 hours, our results show that each lineage harbours a population of cells that do not divide for approximately 8–10 cell cycles after the pulse.

During EdU staining in whole animals, label-retaining cells were also observed in buds that were still attached to the parent (supplementary material Fig. S3A). This was particularly interesting since buds arise from cells that divide in the body column of the parent, followed by tissue displacement in the growing bud. Developing buds also have a high labelling index indicating a higher rate of cell division (Holstein et al., 1991). We also observed that the cells in buds have a high rate of proliferation as seen after a short EdU pulse (supplementary material Fig. S1A). The label-retaining cells in buds comprised of both undifferentiated and differentiated label-retaining cells such as epithelial cells, neurons and nematocytes, with an occasional 1s cell in buds forming at one week (supplementary material Fig. S3B).

### Label-retaining cells can re-enter cell cycle

**Fig. 3.**
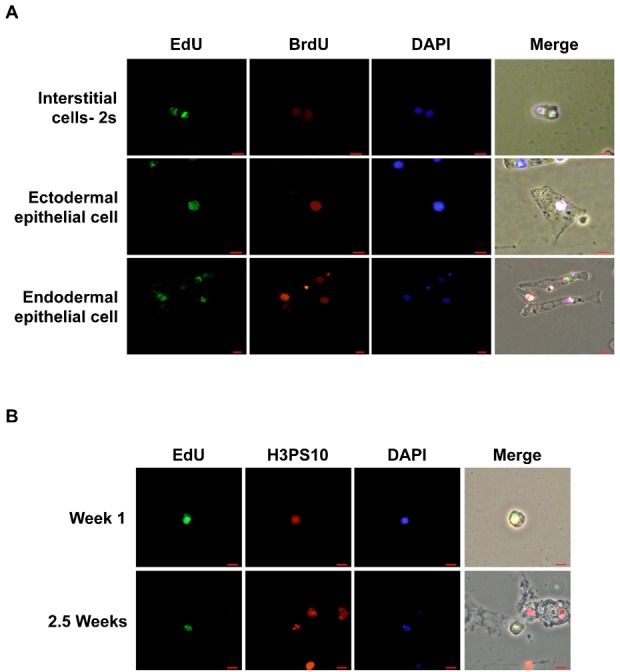
Label-retaining cells can re-enter cell division. (A) Cells in chase were incubated with BrdU for seven days and colocalization of EdU and BrdU was checked. The experiment was performed at one week of chase for interstitial cells and at 3.5 weeks of chase for epithelial cells. Colocalization of EdU and BrdU was observed in cells of all lineages. (B) Colocalization of phosphohistone H3 and EdU was investigated at one week (top panel) and 2.5 weeks (bottom panel) of chase. Scale bars: 10 µm.

We next checked if the label-retaining cells are indeed capable of cell division and not lost from the body column without undergoing cell division. The cell division potential of the slow-cycling cells was investigated by checking their ability to incorporate thymidine analog bromodeoxyuridine (BrdU) during S phase and using anti-phosphohistone H3 (H3P) antibody, which marks cells in the mitotic phase of cell cycle. To investigate the cell division potential of interstitial cells, hydra at three days of chase were incubated with BrdU for five and seven days. To investigate cell division potential of epithelial label-retaining cells, polyps at two weeks of chase were incubated with BrdU for three, five and seven days. At the end of incubation, the polyps were macerated and EdU/BrdU colocalization was investigated. Colocalization of EdU and BrdU was observed in undifferentiated cells of all three lineages, indicating that label-retaining cells were capable of undergoing cell division (Fig. 3A). The figure shows colocalization at day 7 of BrdU incubation. These cells also retained a substantial amount of EdU suggesting that cells had not undergone many cell divisions. Since all label-retaining cells are not expected to be synchronous and therefore all label-retaining cells may not undergo cell division during the pulse with BrdU, only a small fraction of EdU-retaining cells incorporated BrdU but the number of cells with colocalization increased with increasing number of days in BrdU pulse. The cell division potential of label-retaining cells was also confirmed by investigating colocalization of EdU with H3P (Fig. 3B).

### Label-retaining 1s/2s cells localize close to the extracellular matrix

**Fig. 4.**
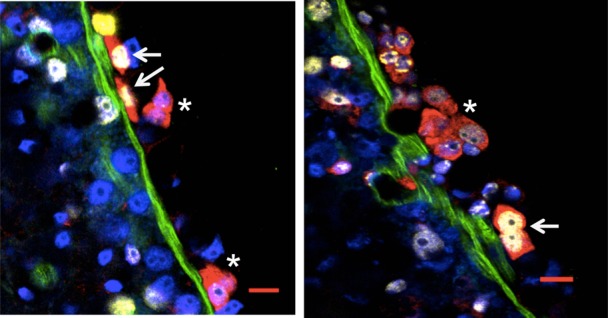
Label-retaining interstitial stem cells localize close to the extracellular matrix. Localization of GFP (red), laminin (green) and EdU-retaining cells (yellow) in cnnos1::GFP lines at one week of chase. Nuclei are stained with DAPI (blue). The label-retaining interstitial stem cells localize close to the extracellular matrix and are marked with arrows while those with partial label are marked with asterisks. Scale bars: 10 µm. Individual images of each fluorophore are shown in supplementary material Fig. S4A,B.

It has been suggested that the niche for multipotent interstitial stem cells is provided by the extracellular matrix (ECM) and the neighbouring epithelial cells (Bosch et al., 2010). The localization of label-retaining 1s/2s cells with respect to the extracellular matrix was investigated using AEP strain expressing GFP under *nanos1* promoter which is highly expressed only in 1s/2s interstitial cells (cnnos1::eGFP) (Hemmrich et al., 2012). As shown in Fig. 4, a number of EdU-retaining 1s/2s cells were in close contact with laminin. The percentage of label-retaining 1s/2s cells in contact with ECM was higher than those which retained partial label indicating cell division. 76% label-retaining 1s/2s cells were close to the ECM, in contrast 42% 1s/2s cells with partial label were in close contact with ECM. These results suggest that the label-retaining cells are predominantly present in close contact with the ECM and the ECM could provide niche for label-retaining interstitial stem cells.

### Slow-cycling cells are resistant to hydroxyurea treatment

**Fig. 5.**
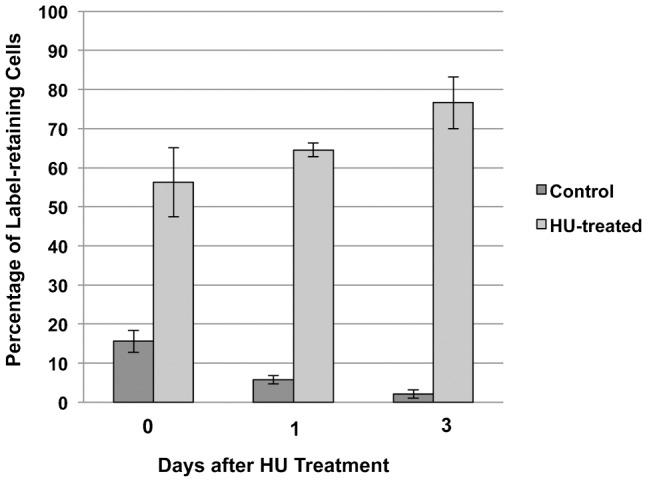
Slow cycling cells are resistant to hydroxyurea (HU) treatment. Hydra were pulsed with EdU for one week, chased for two days and then treated with hydroxyurea for five days. The percentage of EdU retaining interstitial cells (1s and 2s) at the end of hydroxyurea treatment, at one day after hydroxyurea treatment and three days after hydroxyurea treatment is shown. The data represent mean and standard deviation from two independent experiments.

Interstitial cells divide faster than epithelial cells and consequently can be eradicated from hydra by treatments that affect dividing cells, such as hydroxyurea or gamma irradiation (Bode et al., 1976; Fradkin et al., 1978). Slow-cycling cells should be resistant to hydroxyurea treatment since they do not enter cell cycle. To investigate if these cells are indeed resistant to hydroxyurea, hydra at two days of chase were treated with hydroxyurea for five consecutive days. Polyps were macerated at the end of hydroxyurea treatment as well as at one and three days after the hydroxyurea treatment. During these three days the polyps were incubated in hydra medium without hydroxyurea. At the end of hydroxyurea treatment, the number of 1s and 2s interstitial cells reduced by more than 95% as expected (data not shown). However among the interstitial cells present at the end of treatment, a higher percentage of label-retaining 1s and 2s cells was observed in treated polyps as compared to the untreated control (Fig. 5). In polyps treated with hydroxyurea, 56% 1s and 2s cells present at the end of hydroxyurea treatment retained label as compared to 16% in the control polyps that were not treated with hydroxyurea. At one and three days after hydroxyurea treatment, the percentage of label-retaining interstitial cells further increased to 90% in treated cells while the number of label-retaining cells in the control decreased (Fig. 5). The increase in the relative abundance of label-retaining cells was not due to increase in the number of label-retaining cells but was due to decrease in cells without label. These results show that the label-retaining cells are indeed resistant to hydroxyurea treatment.

### Slow-cycling cells pause in G2 phase of cell cycle

**Fig. 6.**
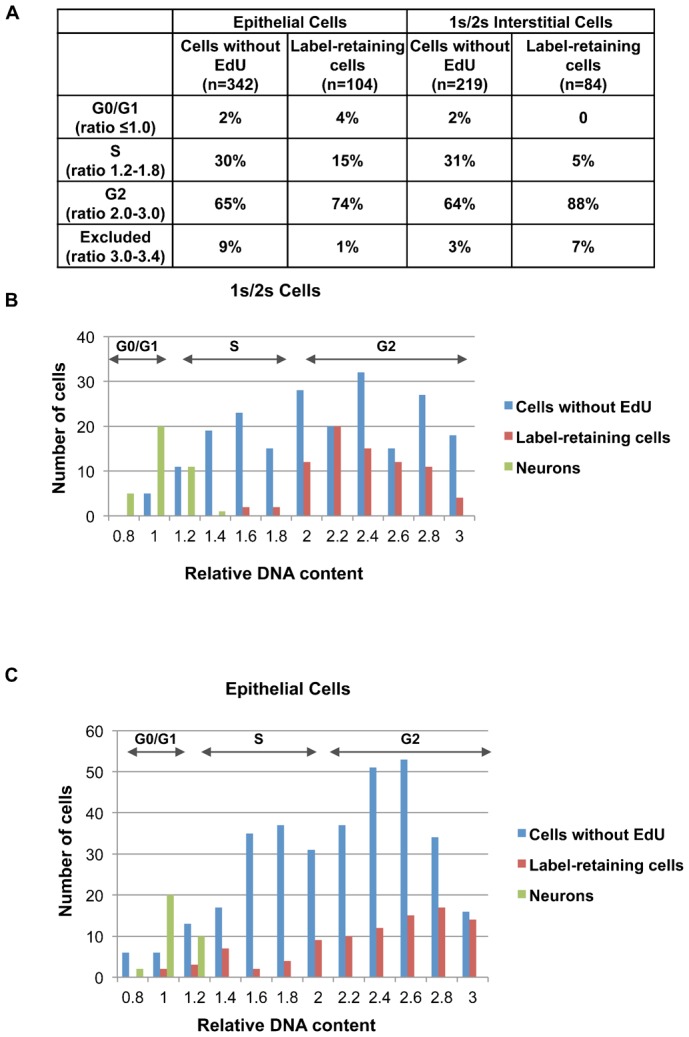
Slow-cycling cells pause in G2 phase. DNA content of cells that retained label and those that did not retain label was measured using DNA content of neurons as standard for 2N DNA content. The experiment was performed at 1 week of chase for interstitial cells and at 3.5 week of chase for epithelial cells. (A) The percentage of label-retaining cells and cells without label in different phases of cell cycle. (B) Cell cycle analysis of 1s/2s cells. (C) Cell cycle analysis of epithelial cells. Label-retaining cells of all lineages were predominantly present in the G2 phase. Data pooled from three independent experiments are shown.

The quiescent stem cells in mammals are held in G0/G1 phase of cell cycle. In hydra it was recently shown that stem cells of interstitial lineages pause at G2 phase (Buzgariu et al., 2014). To check the cell cycle stage in which the slow-cycling cells are held, DNA content of slow-cycling interstitial stem cells was measured by fluorescence microscopy. The experiment was performed at one week of chase for interstitial cells and at 3.5 weeks of chase for epithelial cells. Polyps in chase were macerated, stained for EdU and with DAPI. The DNA content of neurons was taken to be 2N and the DNA content of interstitial and epithelial cells, retaining label and those without EdU, was measured in comparison with neuronal DNA content. As shown in Fig. 6, consistent with the reported cell cycle analysis of 1s/2s cells, 1s/2s cells without EdU were predominantly present in G2 phase with a smaller fraction in G0/G1. The label-retaining cells of interstitial as well as epithelial cells were also predominantly present in G2 phase and the percentage of cells in G2 phase was higher in label-retaining cells than in those without label (Fig. 6A–C), in both interstitial and epithelial lineages.

### Slow-cycling cells are recruited to divide during regeneration

**Fig. 7.**
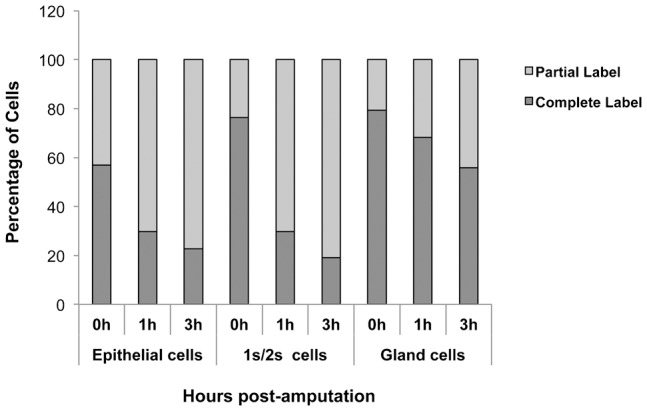
Slow-cycling cells divide during head regeneration. The experiment was performed at one week of chase for interstitial cells and at 2.5–3.5 weeks of chase for epithelial cells. For each time point, 15 regenerating tips (∼0.5 mm) from lower halves that develop into heads were collected. Tips were macerated, stained for EdU and the numbers of cells with complete and partial label were counted. Data pooled from three independent experiments are shown.

Quiescent stem cells in mammals are activated in response to injury and specifically contribute to regenerating tissue. To check if the slow-cycling cells in hydra contribute to head regeneration, midgastric cut was performed. At one and three hours after amputation (hpa), the regenerating tips developing head (∼500 µm) were cut and macerated. This experiment was performed at one week of chase to investigate contribution of interstitial cells and at 2.5–3.5 weeks of chase to investigate the contribution of epithelial cells. The numbers of EdU-retaining cells with complete label and partial label in the macerates were analysed. Control for this experiment was an equivalent region in the body column of polyps in chase; this region was cut and immediately macerated for EdU staining. As shown in Fig. 7, in both epithelial and interstitial slow-cycling cells, there was ∼50% decrease in the number of cells with complete label at 1 hpa. Concomitantly there was an increase in cells with partial label, indicating that the slow-cycling cells had divided during this time. The cells with complete label did not further decrease substantially at 3 hpa (Fig. 7). There was also a small decrease in number of gland cells with complete label and a concomitant increase in gland cells with partial label but this change in the number of label-retaining cells was not as significant as that in epithelial and interstitial cells. The slow-cycling cells were thus recruited to undergo cell division within first hour of regeneration.

## DISCUSSION

Studies carried out on cell dynamics in hydra suggest that the hydra body column largely consists of stem cells which divide continuously (Bosch et al., 2010; Hobmayer et al., 2012). Here, we report heterogeneity in the cell cycle frequency of hydra stem cells and show that there is a sub-population of stem cells that divides infrequently. Slow-cycling stem cells from all the three lineages; ectodermal epithelial lineage, endodermal epithelial lineage and interstitial lineage can remain undivided for approximately 8–10 cell division cycles. These cells can re-enter cell cycle, are resistant to agents that cause DNA damage and are also present in buds. The slow-cycling cells are predominantly in G2 phase of cell cycle. Most importantly, similar to mammalian quiescent stem cells, the slow-cycling stem cells are activated to undergo cell division during head regeneration, within an hour after amputation.

Previous studies have reported heterogeneity in cell cycle time across different cell types within interstitial lineage (Holstein and David, 1990). Variation in the cell cycle of different interstitial cells is mainly due to variations in the length of G2. In 1s/2s interstitial cells the G2 phase is 4–22 hr, it is 3–4 h in 4s/8s/16s and that of sex-specific stem cells about three days (Campbell and David, 1974; Holstein and David, 1990). The S-phase of all cells of interstitial origin is 11–12 h and the G1 is approximately 1 h. The cell cycle of interstitial cells shortens under conditions where the number of interstitial cells in hydra decreases and faster cell division is required to regain the required number of interstitial cells (Holstein and David, 1990). No heterogeneity has been reported in epithelial cells with respect to their cell cycle. Anecdotal evidence does exist indicating that [^3^H] thymidine or BrdU labelling does not lead to complete labelling of all cells in hydra but the general consensus in literature has been that all stem cells within each lineage are functionally equivalent. No study has been carried out so far to explore this heterogeneity and its functional significance. Our studies show heterogeneity within the cell cycle of the undifferentiated cells of epithelial lineages and the multipotent 1s and 2s cells of interstitial lineage, rather than across different cell types within interstitial lineage as described earlier. The interstitial stem cells remain undivided for about 10 days while the epithelial cells remain undivided for ∼4–5 weeks. Considering the cell cycle time of 18–27 hours in interstitial stem cells and 3–4 days in epithelial cells, the cells do not divide for approximately 8–10 cell cycles. Slow-cycling cells described in this study are likely not the germline stem cells since the strain used in this study, *H. magnipapillata* grows as asexual polyp and no gametogenesis has been observed in this strain over last four years in our laboratory. We also did not see cluster-like arrangement of slow-cycling cells as is seen in the germ cells of hydra (Nishimiya-Fujisawa and Kobayashi, 2012). Gland cells, though differentiated, also undergo cell division with cell cycle time of 64–83 hours (Schmidt and David, 1986). We see large population of label-retaining gland cells. Many of these gland cells possibly retained label due to terminal differentiation. However some of these gland cells indeed divide during long chase as seen by colocalization of BrdU and EdU in these cells even at 3 weeks of chase, indicating the presence of slow-cycling gland cells (data not shown). The slow-cycling interstitial cells were resistant to hydroxyurea treatment, as expected. Presence of such resistant cells also explains observations from previous studies where spontaneous recovery of cells of interstitial lineage was observed in epithelial polyps post-hydroxyurea treatment (Bode et al., 1976; Technau and Holstein, 1996). These previous studies suggest presence of hydroxyurea-resistant interstitial stem cells and our studies show that this recovery of interstitial lineage is due to slow-cycling interstitial cells.

We did not observe any difference in the morphology of slow-cycling cells as compared to cells that were dividing continuously. The slow-cycling cells also do not localise to any specific location and are distributed throughout the body column. This suggests that the niche for slow cycling cells is not position-dependent but is perhaps provided by the local environment. It has been hypothesized that the niche for interstitial stem cells could be provided by the ECM and the neighbouring epithelial cells. The label-retaining 1s/2s cells indeed localized close to the ECM. Whether proximity of these cells to ECM indeed regulates their cell cycle potential or pluripotency remains to be seen.

The slow-cycling cells also were observed in buds of hydra in chase. Buds arise from dividing cells in the body column that are displaced into buds. Cells in buds are highly proliferative and a short EdU pulse labels a large number of cells in buds (Holstein et al., 1991). A small number of slow-cycling cells from parent hydra migrate into the buds without undergoing cell division, which suggests that buds also acquire cells that have undergone less number of cell divisions and have possibly accumulated less number of errors. Tissue movement of epithelial cells along with the extracellular matrix has been reported in hydra (Aufschnaiter et al., 2011) and is likely responsible for movement of slow-cycling cells in the buds.

Our studies also show that the label-retaining cells are capable of undergoing cell division, as seen by incorporation of BrdU and H3P staining. Since the cells do not divide synchronously, it is not possible to comment on cell cycle time of the label-retaining cells. Importantly, our studies show that the slow-cycling cells of all lineages are recruited to divide during the first hour of head regeneration. These cells divide during the first hour after amputation, in the regenerating head. There is no further recruitment of slow-cycling cells at later times like 3 hpa and 6 hpa. Cells in hydra being highly proliferative can get labelled with thymidine analogs in a time as short as one hour (Plickert and Kroiher, 1988). It has also been shown previously that cells in the regenerating tip undergo cell division (Chera et al., 2009). This study has shown that in a head-regenerating tip, the interstitial cells localized in region 2 of the regenerating tip (between 100–200 µm from the regenerating tip) undergo cell division between 0–2 hpa but not between 2–4 hpa. We have looked at the entire region corresponding to ∼500 µm of the head-regenerating tip, where we see both interstitial and epithelial slow-cycling cells undergoing cell division. The difference in the results reported earlier (Chera et al., 2009) and our study could be due to difference in the regions of head-regenerating tip investigated in the two studies. Moreover our studies were aimed at investigating the cell cycle kinetics of a subset of cells consisting of slow-cycling cells.

It has been reported by Holstein and co-workers that the labelling index of the regenerating tip in epithelial hydra decreases in first 12 h of regeneration but increases around 30 h (Holstein et al., 1991). This is in contrast to the reports by Chera and coworkers (Chera et al., 2009) and our report in this manuscript. This variation could either be due to the difference in the system used or due to the differences in the regenerating tissue that was used for these studies. Holstein and coworkers have used epithelial hydra and in the cell cycle kinetics in these animals could be different from that in normal animals with interstitial cells. The region of regenerating tip used for maceration was also different in this study (1/5th of regenerating hydra as against ∼500 µm of regenerating tip in our study). The large number of cells from region just below regenerating tip could have masked the differences in the actual regenerating tip. Recruiting such slow-cycling cells that have remained undivided would ensure that the newly regenerated hydra has cells with better genomic integrity. All slow-cycling cells from the regenerating tip do not however divide during regeneration (Fig. 7). It is possible that the slow-cycling cells closer to the site of injury, and not the entire macerated tip, are recruited during cell division. The ability of slow-cycling cells from hydra to divide rapidly in response to injury is very similar to that of quiescent stem cells from adult mammalian tissues.

Interestingly, the slow-cycling cells were paused in G2 phase of cell cycle and not in G0 as in the case of mammalian cells. It has been recently shown that the interstitial stem cells in hydra pause in G2 phase before undergoing next round of cell cycle (Buzgariu et al., 2014). Consistent with this report, we find that slow cycling cells of not only interstitial lineage but also of epithelial lineage are present predominantly in G2. The percentage of slow-cycling cells in G2 phase was even higher than those that entered cell cycle (Fig. 6). Both hydra and mammalian cells regulate the frequency with which cells divide; however, the molecular mechanism of this regulation appears to be different among these two groups of organisms.

While we find ∼2% cells of all lineages to be slow-cycling cells, it is possible that this study has underestimated the actual number of slow-cycling cells. This study assumes that all cells underwent at least one cell division during pulse. Some cells may have remained quiescent during pulse and may have been missed in subsequent chase. A comparison of one week pulse and four week pulse indeed showed that the labelling index increased with increasing time of pulse (Table 1). Pulse beyond four weeks was not done since the thymidine analogs can also have adverse effects (Kaufman and Davidson, 1978) and developmental defects were indeed observed during longer pulse with EdU, as described in results. Correct estimation of the number of slow-cycling cells needs to be performed using tools that are independent of labelled nucleotides, such as by generation of transgenic lines expressing H2B-GFP under an inducible promoter. Histone H2B is associated stably with nucleosomes and is replaced during mitosis. In an inducible system for H2B tagged with GFP, H2B-GFP can be induced during a specific window, expression can then be switched off and retention of H2B-GFP indicative of undivided cells can be monitored over several cell cycles. It is therefore an excellent marker for detection of label-retaining cells. This marker has been used to identify quiescent cells and trace their lineage in hair follicles (Tumbar et al., 2004), hematopoietic cells (Foudi et al., 2009) and intestinal epithelial cells (Buczacki et al., 2013). Such tools are currently not available for this model organism.

In the last decade, identification of specific stem cell markers has allowed lineage tracing of stem cells. These experiments reveal the presence of two distinct stem cell populations; a population that divides continuously to maintain homeostasis and one that divides specifically during regeneration. Such distinct populations have been observed in intestinal crypts and hematopoietic stem cells. In intestinal crypts, stem cells expressing *Lgr5* divide continuously to maintain normal homeostasis while a population of *Bmi* expressing stem cells divides only during injury-induced regeneration (Tian et al., 2011; Yan et al., 2012). Hematopoietic stem cells have population of quiescent stem cells that divides very rarely (once in 145 days) but can proliferate actively in response to injury (Foudi et al., 2009; Wilson et al., 2008). Such distinct pools of stem cells are however not well-defined in stem cell systems such as hair follicle (Fuchs, 2009; Ito et al., 2005; Tumbar et al., 2004), satellite cells in muscles (Montarras et al., 2013) or limbal stem cells (Cotsarelis et al., 1989). At the moment it is not possible to comment on whether hydra harbours separate pools of stem cells to maintain homeostasis and regeneration or whether the same pool of stem cells coordinates both processes.

Paradoxically, while quiescence is meant to prevent DNA damage in stem cells, long term quiescence itself can lead to DNA damage. In aging mice, quiescent hematopoietic stem cells accumulate DNA damage as seen by accumulation of H2AX foci (Rossi et al., 2007). This situation arises due to lack of DNA damage checkpoints that operate during cell cycle, leading to slow accumulation of DNA damage over long periods of quiescence. The slow-cycling cells in hydra do not remain quiescent indefinitely but divide once approximately every eight cell cycles. This situation may possibly be more favourable than maintaining two separate pools of stem cells since it would help prevent DNA damage associated with long periods of quiescence.

Stem cells are an essential feature of all multicellular organisms, without which multicellular organisms will not exist. The degree of similarity between stem cells in early multicellular organisms like cnidarians and stem cells in higher organisms is not known. Some markers such as Oct4 family gene *polynem*, *piwi*, *nanos* and *vasa* are specifically expressed in cnidarian stem cells but whether their functional role is similar to their homologs in mammals is not yet clear (Millane et al., 2011; Watanabe et al., 2009). Presence of cells that remain undivided for several cell cycles suggests that stem cells in hydra share at least one characteristic of cell cycle regulation with mammalian stem cells, namely the ability to retain stem cells in slow-cycling state. A key question now is to understand the molecular mechanisms that regulate this process and determine if slow-cycling stem cells in hydra differ from rest of the stem cells in terms of their ability to self-renew or differentiate. To conclude, our studies show that hydra possesses a population of slow-cycling stem cells that is recruited during regeneration, a characteristic it shares with mammalian quiescent stem cells. These studies show that the ability to maintain stem cell populations with heterogeneity in the frequency of cell cycle arose early in the evolution of multicellular organisms.

## Supplementary Material

Supplementary Material

## References

[b1] Aufschnaiter R. Zamir E. A. Little C. D. Özbek S. Münder S. David C. N. Li L. Sarras M. P. Jr and Zhang X. (2011). In vivo imaging of basement membrane movement: ECM patterning shapes Hydra polyps. J. Cell Sci. 124, 4027–4038 10.1242/jcs.087239 22194305 PMC3244984

[b2] Bode H. R. Flick K. M. Smith G. S. (1976). Regulation of interstitial cell differentiation in Hydra attenuata. I. Homeostatic control of interstitial cell population size. J. Cell Sci. 20, 29–46. 1249123 10.1242/jcs.20.1.29

[b3] Bosch T. C. (2003). Ancient signals: peptides and the interpretation of positional information in ancestral metazoans. Comp. Biochem. Physiol. 136B, 185–196 10.1016/S1096-4959(03)00226-4 14529745

[b4] Bosch T. C. Anton-Erxleben F. Hemmrich G. Khalturin K. (2010). The Hydra polyp: nothing but an active stem cell community. Dev. Growth Differ. 52, 15–25 10.1111/j.1440-169X.2009.01143.x 19891641

[b5] Buczacki S. J. Zecchini H. I. Nicholson A. M. Russell R. Vermeulen L. Kemp R. Winton D. J. (2013). Intestinal label-retaining cells are secretory precursors expressing Lgr5. Nature 495, 65–69 10.1038/nature11965 23446353

[b6] Buzgariu W. Crescenzi M. Galliot B. (2014). Robust G2 pausing of adult stem cells in Hydra. Differentiation 87, 83–99 10.1016/j.diff.2014.03.001 24703763

[b7] Campbell R. D. (1973). Vital marking of single cells in developing tissues: India ink injection to trace tissue movements in hydra. J. Cell Sci. 13, 651–661. 4129642 10.1242/jcs.13.3.651

[b8] Campbell R. D. David C. N. (1974). Cell cycle kinetics and development of Hydra attenuata. II. Interstitial cells. J. Cell Sci. 16, 349–358. 4448825 10.1242/jcs.16.2.349

[b9] Cheng T. Rodrigues N. Dombkowski D. Stier S. Scadden D. T. (2000a). Stem cell repopulation efficiency but not pool size is governed by p27(kip1). Nat. Med. 6, 1235–1240 10.1038/81335 11062534

[b10] Cheng T. Rodrigues N. Shen H. Yang Y. Dombkowski D. Sykes M. Scadden D. T. (2000b). Hematopoietic stem cell quiescence maintained by p21cip1/waf1. Science 287, 1804–1808 10.1126/science.287.5459.1804 10710306

[b11] Chera S. Ghila L. Dobretz K. Wenger Y. Bauer C. Buzgariu W. Martinou J. C. Galliot B. (2009). Apoptotic cells provide an unexpected source of Wnt3 signaling to drive hydra head regeneration. Dev. Cell 17, 279–289 10.1016/j.devcel.2009.07.014 19686688

[b12] Cheshier S. H. Morrison S. J. Liao X. Weissman I. L. (1999). In vivo proliferation and cell cycle kinetics of long-term self-renewing hematopoietic stem cells. Proc. Natl. Acad. Sci. USA 96, 3120–3125 10.1073/pnas.96.6.3120 10077647 PMC15905

[b13] Cheung T. H. Rando T. A. (2013). Molecular regulation of stem cell quiescence. Nat. Rev. Mol. Cell Biol. 14, 329–340 10.1038/nrm3591 23698583 PMC3808888

[b14] Cotsarelis G. Cheng S. Z. Dong G. Sun T. T. Lavker R. M. (1989). Existence of slow-cycling limbal epithelial basal cells that can be preferentially stimulated to proliferate: implications on epithelial stem cells. Cell 57, 201–\209 10.1016/0092-8674(89)90958-6 2702690

[b15] David C. N. (1973). A quantitative method for maceration of *Hydra* tissue. Wilhelm Roux Arch. Entwickl. Mech. Org. 171, 259–268 10.1007/BF00577724 28304607

[b16] David C. N. (2012). Interstitial stem cells in Hydra: multipotency and decision-making. Int. J. Dev. Biol. 56, 489–497 10.1387/ijdb.113476cd 22689367

[b17] David C. N. Campbell R. D. (1972). Cell cycle kinetics and development of Hydra attenuata. I. Epithelial cells. J. Cell Sci. 11, 557–568. 5076361 10.1242/jcs.11.2.557

[b18] Foudi A. Hochedlinger K. Van Buren D. Schindler J. W. Jaenisch R. Carey V. Hock H. (2009). Analysis of histone 2B-GFP retention reveals slowly cycling hematopoietic stem cells. Nat. Biotechnol. 27, 84–90 10.1038/nbt.1517 19060879 PMC2805441

[b19] Fradkin M. Kakis H. Campbell R. D. (1978). Effect of gamma irradiation of hydra: elimination of interstitial cells from viable hydra. Radiat. Res. 76, 187–197 10.2307/3574938 734045

[b20] Fuchs E. (2009). The tortoise and the hair: slow-cycling cells in the stem cell race. Cell 137, 811–819 10.1016/j.cell.2009.05.002 19490891 PMC2716122

[b21] Fuchs E. Horsley V. (2011). Ferreting out stem cells from their niches. Nat. Cell Biol. 13, 513–518 10.1038/ncb0511-513 21540847 PMC3289407

[b22] Gierer A. Berking S. Bode H. David C. N. Flick K. Hansmann G. Schaller H. Trenkner E. (1972). Regeneration of hydra from reaggregated cells. Nat. New Biol. 239, 98–101 10.1038/newbio239098a0 4507522

[b23] Hemmrich G. Khalturin K. Boehm A. M. Puchert M. Anton-Erxleben F. Wittlieb J. Klostermeier U. C. Rosenstiel P. Oberg H. H. Domazet-Loso T. (2012). Molecular signatures of the three stem cell lineages in hydra and the emergence of stem cell function at the base of multicellularity. Mol. Biol. Evol. 29, 3267–3280 10.1093/molbev/mss134 22595987

[b24] Hobmayer B. Jenewein M. Eder D. Eder M. K. Glasauer S. Gufler S. Hartl M. Salvenmoser W. (2012). Stemness in Hydra - a current perspective. Int. J. Dev. Biol. 56, 509–517 10.1387/ijdb.113426bh 22689357

[b25] Holstein T. W. David C. N. (1990). Cell cycle length, cell size, and proliferation rate in hydra stem cells. Dev. Biol. 142, 392–400 10.1016/0012-1606(90)90360-U 2257972

[b26] Holstein T. W. Hobmayer E. David C. N. (1991). Pattern of epithelial cell cycling in hydra. Dev. Biol. 148, 602–611 10.1016/0012-1606(91)90277-A 1743403

[b27] Illa-Bochaca I. Montuenga L. M. (2006). The regenerative nidi of the locust midgut as a model to study epithelial cell differentiation from stem cells. J. Exp. Biol. 209, 2215–2223 10.1242/jeb.02249 16709922

[b28] Ito M. Liu Y. Yang Z. Nguyen J. Liang F. Morris R. J. Cotsarelis G. (2005). Stem cells in the hair follicle bulge contribute to wound repair but not to homeostasis of the epidermis. Nat. Med. 11, 1351–1354 10.1038/nm1328 16288281

[b29] Kaufman E. R. Davidson R. L. (1978). Bromodeoxyuridine mutagenesis in mammalian cells: mutagenesis is independent of the amount of bromouracil in DNA. Proc. Natl. Acad. Sci. USA 75, 4982–4986 10.1073/pnas.75.10.4982 283408 PMC336246

[b30] Krishna S. Nair A. Cheedipudi S. Poduval D. Dhawan J. Palakodeti D. Ghanekar Y. (2013). Deep sequencing reveals unique small RNA repertoire that is regulated during head regeneration in Hydra magnipapillata. Nucleic Acids Res. 41, 599–616 10.1093/nar/gks1020 23166307 PMC3592418

[b31] Martínez D. E. (1998). Mortality patterns suggest lack of senescence in hydra. Exp. Gerontol. 33, 217–225 10.1016/S0531-5565(97)00113-7 9615920

[b32] Millane R. C. Kanska J. Duffy D. J. Seoighe C. Cunningham S. Plickert G. Frank U. (2011). Induced stem cell neoplasia in a cnidarian by ectopic expression of a POU domain transcription factor. Development 138, 2429–2439 10.1242/dev.064931 21610024

[b33] Montarras D. L'honoré A. Buckingham M. (2013). Lying low but ready for action: the quiescent muscle satellite cell. FEBS J. 280, 4036–4050 10.1111/febs.12372 23735050

[b34] Morris R. J. Potten C. S. (1999). Highly persistent label-retaining cells in the hair follicles of mice and their fate following induction of anagen. J. Invest. Dermatol. 112, 470–475 10.1046/j.1523-1747.1999.00537.x 10201531

[b35] Nishimiya-Fujisawa C. Kobayashi S. (2012). Germline stem cells and sex determination in Hydra. Int. J. Dev. Biol. 56, 499–508 10.1387/ijdb.123509cf 22689373

[b36] Plickert G. Kroiher M. (1988). Proliferation kinetics and cell lineages can be studied in whole mounts and macerates by means of BrdU/anti-BrdU technique. Development 103, 791–794. 3073936 10.1242/dev.103.4.791

[b37] Rossi D. J. Bryder D. Seita J. Nussenzweig A. Hoeijmakers J. Weissman I. L. (2007). Deficiencies in DNA damage repair limit the function of haematopoietic stem cells with age. Nature 447, 725–729 10.1038/nature05862 17554309

[b38] Rossi D. J. Jamieson C. H. Weissman I. L. (2008). Stems cells and the pathways to aging and cancer. Cell 132, 681–696 10.1016/j.cell.2008.01.036 18295583

[b39] Sarras M. P. Jr (2012). Components, structure, biogenesis and function of the Hydra extracellular matrix in regeneration, pattern formation and cell differentiation. Int. J. Dev. Biol. 56, 567–576 10.1387/ijdb.113445ms 22689358

[b40] Schmidt T. David C. N. (1986). Gland cells in Hydra: cell cycle kinetics and development. J. Cell Sci. 85, 197–215. 3539952 10.1242/jcs.85.1.197

[b41] Tan J. Xu M. Zhang K. Wang X. Chen S. Li T. Xiang Z. Cui H. (2013). Characterization of hemocytes proliferation in larval silkworm, Bombyx mori. J. Insect Physiol. 59, 595–603 10.1016/j.jinsphys.2013.03.008 23557681

[b42] Technau U. Holstein T. W. (1996). Phenotypic maturation of neurons and continuous precursor migration in the formation of the peduncle nerve net in Hydra. Dev. Biol. 177, 599–615 10.1006/dbio.1996.0189 8806835

[b43] Tian H. Biehs B. Warming S. Leong K. G. Rangell L. Klein O. D. de Sauvage F. J. (2011). A reserve stem cell population in small intestine renders Lgr5-positive cells dispensable. Nature 478, 255–259 10.1038/nature10408 21927002 PMC4251967

[b44] Tumbar T. Guasch G. Greco V. Blanpain C. Lowry W. E. Rendl M. Fuchs E. (2004). Defining the epithelial stem cell niche in skin. Science 303, 359–363 10.1126/science.1092436 14671312 PMC2405920

[b45] Verdoodt F. Willems M. Mouton S. De Mulder K. Bert W. Houthoofd W. Smith J. 3rd Ladurner P. (2012). Stem cells propagate their DNA by random segregation in the flatworm Macrostomum lignano. *PLoS ONE* 7, e30227 10.1371/journal.pone.0030227 PMC3261893 22276162

[b46] Watanabe H. Hoang V. T. Mättner R. Holstein T. W. (2009). Immortality and the base of multicellular life: Lessons from cnidarian stem cells. Semin. Cell Dev. Biol. 20, 1114–1125 10.1016/j.semcdb.2009.09.008 19761866

[b47] Wilson A. Laurenti E. Oser G. van der Wath R. C. Blanco-Bose W. Jaworski M. Offner S. Dunant C. F. Eshkind L. Bockamp E. (2008). Hematopoietic stem cells reversibly switch from dormancy to self-renewal during homeostasis and repair. Cell 135, 1118–1129 10.1016/j.cell.2008.10.048 19062086

[b48] Wittlieb J. Khalturin K. Lohmann J. U. Anton-Erxleben F. Bosch T. C. (2006). Transgenic Hydra allow in vivo tracking of individual stem cells during morphogenesis. Proc. Natl. Acad. Sci. USA 103, 6208–6211 10.1073/pnas.0510163103 16556723 PMC1458856

[b49] Yan K. S. Chia L. A. Li X. Ootani A. Su J. Lee J. Y. Su N. Luo Y. Heilshorn S. C. Amieva M. R. (2012). The intestinal stem cell markers Bmi1 and Lgr5 identify two functionally distinct populations. Proc. Natl. Acad. Sci. USA 109, 466–471 10.1073/pnas.1118857109 22190486 PMC3258636

