# Supplementary material for: Slow-cycling stem cells in hydra contribute to head regeneration
Source: Biol Open. 2014 Nov 28;3(12):1236–44. doi: 10.1242/bio.201410512 (PMC4265762; doi:10.1242/bio.201410512)
Supplement: Supplementary Material [file supp_3_12_1236__index.html]

Slow-cycling stem cells in hydra contribute to head regeneration — Slow-cycling stem cells in hydra contribute to head regeneration — Supplementary Material 

# Slow-cycling stem cells in hydra contribute to head regeneration

## bio.201410512 Supplementary Material

**Files in this Data Supplement:**

- Supplementary Material - Niraimathi Govindasamy et al. doi: 10.1242/bio.201410512
