# Supplementary material for: Slow-cycling stem cells in hydra contribute to head regeneration
Source: Biol Open. 2014 Nov 28;3(12):1236–44. doi: 10.1242/bio.201410512 (PMC4265762; doi:10.1242/bio.201410512)
Supplement: Supplementary Material [file supp_bio.201410512_bio.201410512-s1.pdf]

Biology Open (2014) 000, 1–9 doi:10.1242/bio.201410512

Supplementary Material

Niraimathi Govindasamy et al. doi: 10.1242/bio.201410512

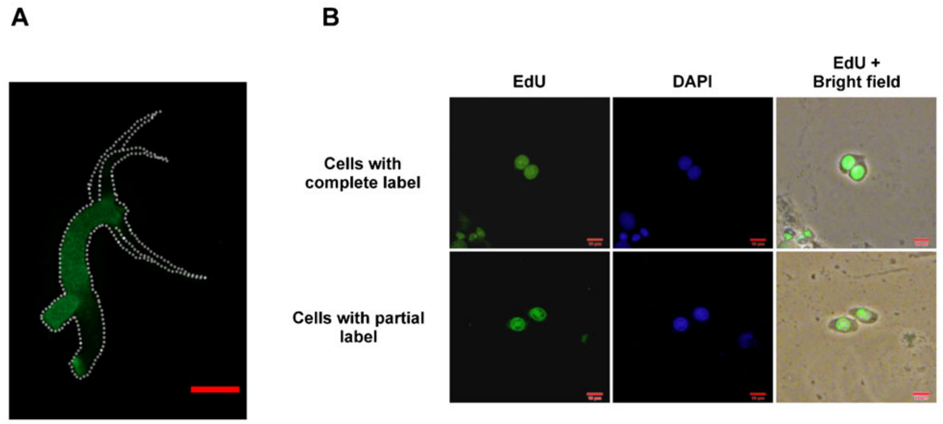

Fig. S1. (A) EdU efficiently labels dividing cells in hydra. The figure shows EdU uptake after three hour EdU pulse. EdU staining is observed in the body column where dividing cells are present. The staining at the tip of the foot is nonspecific. (B) Identification of cells with complete and partial label. Macerates were stained for EdU followed by incubation with DAPI to stain nuclear DNA. The cells in the top panel show EdU staining that completely covers DAPI staining, cells with such pattern were considered to be label-retaining cells. The cells in the bottom panel show punctate EdU staining that does not completely encompass nuclear DNA, such pattern of staining indicates that cells have undergone cell division and these were not considered as label-retaining cells. Scale bars: 1 mm (A), 10 μm (B).

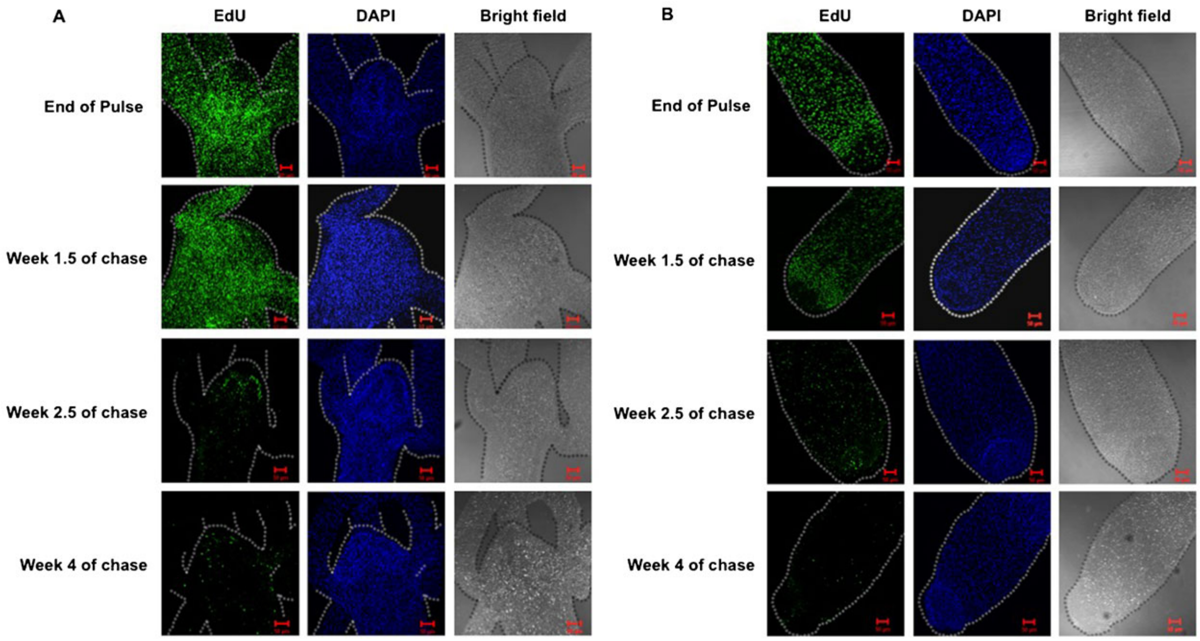

Fig. S2. Label-retaining cells in whole mounts of hydra at different times of chase. Hydra were pulsed with EdU for one week and then cultured without EdU for four weeks. (A) Label-retaining cells in hypostome at different times of chase. (B) Label-retaining cells in foot at different times of chase. Scale bars: 50 μm.

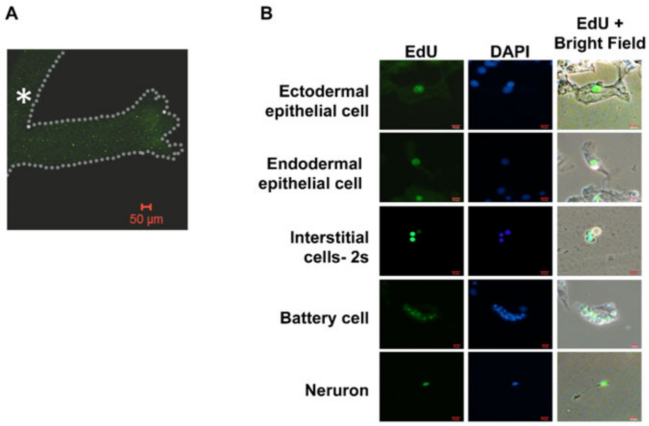

Fig. S3. Identification of label-retaining cells in buds. (A) Whole mount of developing bud showing label-retaining cells at the end of three weeks of chase. Body column of the parent hydra is marked by an asterisk. (B) Buds from hydra in chase were macerated and stained for EdU as described. The battery cell and 2s interstitial cells were seen at the end of 1.5 weeks. Ectodermal and endodermal epithelial cells and nerve cells were observed at the end of four weeks. Scale bars: 50 µm (A), 10 µm (B).

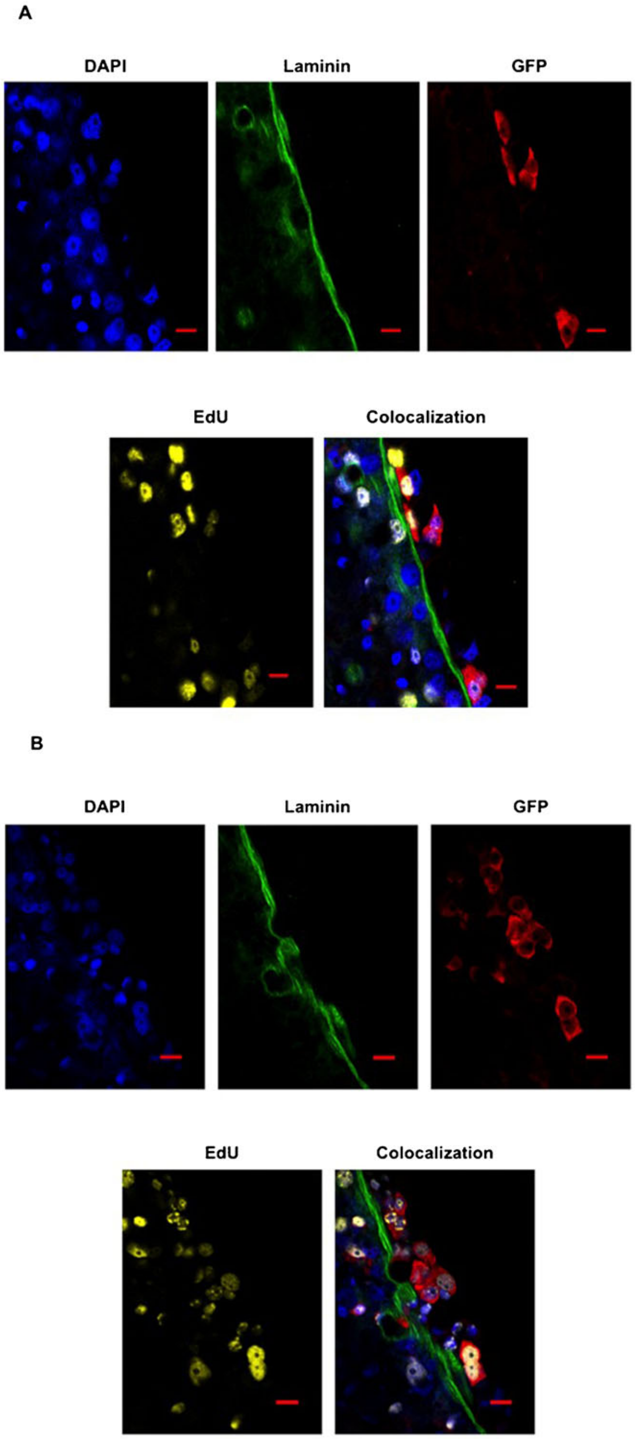

Fig. S4. Label-retaining interstitial stem cells localize close to the extracellular matrix. Signal from individual fluorophores from Fig. 4. (A) Individual fluorophores from the panel on the left of Fig. 4. (B) Individual fluorophores from the panel on the right of Fig. 4. GFP is shown in red, laminin in green and EdU in yellow. Nuclei are stained with DAPI (blue). Scale bars: 10 µm.
